# Climate risk index for Italy

**DOI:** 10.1098/rsta.2017.0305

**Published:** 2018-04-30

**Authors:** Jaroslav Mysiak, Silvia Torresan, Francesco Bosello, Malcolm Mistry, Mattia Amadio, Sepehr Marzi, Elisa Furlan, Anna Sperotto

**Affiliations:** Centro Euro-Mediterraneo sui Cambiamenti Climatici and Università Ca' Foscari, Venezia Porto Marghera, Italy

**Keywords:** climate risk index, vulnerability, adaptive capacity, indicator-based approach, Italy

## Abstract

We describe a climate risk index that has been developed to inform national climate adaptation planning in Italy and that is further elaborated in this paper. The index supports national authorities in designing adaptation policies and plans, guides the initial problem formulation phase, and identifies administrative areas with higher propensity to being adversely affected by climate change. The index combines (i) climate change-amplified hazards; (ii) high-resolution indicators of exposure of chosen economic, social, natural and built- or manufactured capital (MC) assets and (iii) vulnerability, which comprises both present sensitivity to climate-induced hazards and adaptive capacity. We use standardized anomalies of selected extreme climate indices derived from high-resolution regional climate model simulations of the EURO-CORDEX initiative as proxies of climate change-altered weather and climate-related hazards. The exposure and sensitivity assessment is based on indicators of manufactured, natural, social and economic capital assets exposed to and adversely affected by climate-related hazards. The MC refers to material goods or fixed assets which support the production process (e.g. industrial machines and buildings); Natural Capital comprises natural resources and processes (renewable and non-renewable) producing goods and services for well-being; Social Capital (SC) addressed factors at the individual (people's health, knowledge, skills) and collective (institutional) level (e.g. families, communities, organizations and schools); and Economic Capital (EC) includes owned and traded goods and services. The results of the climate risk analysis are used to rank the subnational administrative and statistical units according to the climate risk challenges, and possibly for financial resource allocation for climate adaptation.

This article is part of the theme issue ‘Advances in risk assessment for climate change adaptation policy’.

## 1. Introduction

Climate change is both an unprecedented and a defining environmental challenge of our times, compelling bold and urgent actions simultaneously on many fronts. Climate change adaptation and disaster-resilience planning contribute to transforming world economies along sustainable development pathways [1]. A thorough understanding of climate risks is a catalyst for awareness and action. In a world of constrained budgets, assessing the risks of climate change is ineludible for deciding how to employ resources and in what policy mix. Climate risk assessment is one of the pillars of the review assessment conducted by the Intergovernmental Panel on Climate Change (IPCC). Therein climate risk is portrayed as being composed of hazard, exposure and vulnerability, ‘providing a basis for judgements about the level of climate change at which risks become dangerous' [2, p. 61].

The importance of climate change risk assessment emerges in many policy contexts. For instance, the 2013 EU strategy on adaptation to climate change [3], a top-priority of which is to ‘enhance the preparedness and capacity of all governance levels to respond to the impacts of climate change’, introduces tangible actions to bridge the knowledge gaps. Action 4 stresses a commitment to ‘promote EU-wide vulnerability assessments, by taking into account, among other things, the cross-sectoral EU overview of natural and man-made risks that it will produce’ [3]. National Adaptation Strategies include assessments evaluating the most up-to-date scientific information on potential climate change impacts and/or stress the need to improve that information with appropriate risk assessments.

The concept of risk, hazard and vulnerability have been interpreted in different ways, reflecting the evolution of a variety of scientific disciplines in the fields of climate change adaptation (CCA) and disaster risk reduction (DRR) [4–7]. While the DRR community has historically been more concerned with risk (as a combination of the probability of hazards and their negative consequences) [8]; the CCA community under the IPCC guidance has traditionally put more emphasis on vulnerability [9–12], denoted as a function of exposure, sensitivity and adaptive capacity (AC) [13–17]. Only recently have the IPCC Special Report on Managing the Risks of Extreme Events and Disasters to Advance Climate Change (SREX) [18] and the Fifth Assessment Report (AR5) moved towards a risk-centred framework [19,20].

The conceptual framework of climate vulnerability and risk draws on these reconciled concepts and definitions [21,22]. The electronic supplementary material, table S1, explains more in depth the differences in the above-mentioned frameworks. The key components of the climate risk index (CRI) in this paper are hazard (H), i.e. the potential occurrence of a damaging physical event related to climate trends or climate extremes; exposure (E), i.e. the presence of environmental, social or economic elements in places that could be adversely affected; and vulnerability (V), i.e. the predisposition to be adversely affected, comprising a variety of concepts including sensitivity or susceptibility to harm and the lack of capacity to cope and adapt [20].

As a part of both the AR5 and the earlier AR4 frameworks (see also the electronic supplementary material, table S1), vulnerability is a multi-dimensional concept which refers both to sensitivity and AC. Sensitivity is defined in the IPCC AR4 as ‘the degree to which a system is affected, either adversely or beneficially, by climate variability or change’ and is addressed in various ways, depending on geographical scale, modelling tools, policy needs, data availability and local capacity. Sensitivity is a function of hazard intensity and the properties of the exposed elements [23–25], or approximated using a set of indicators [26–30].

AC [31] denotes ‘the ability of systems, institutions, humans and other organisms to adjust to potential damage, to take advantage of opportunities, or to respond to consequences’ [32]. AC refers to capabilities, resources and institutions driving adoption of adaptation strategies [33,34] and implementation of effective action [13]. The commonly used indicators of AC are linked to economic wealth, technology, information and skills, infrastructure, institutions and equity [12,35], demographic structure, interconnectivity and natural resource use [34].

To assess vulnerability, the underlying physical ecosystem features, their interconnections, and also the human factors such as the economy, institutions, infrastructure and related land use should be investigated and modelled [17,36–38]. Various vulnerability assessments have been conducted at global [39–42], national and regional scales [43–46]. Some studies have addressed vulnerability at the EU-wide context, e.g. the European Observation Network Territorial Development and Cohesion (ESPON) [12], the European Environment Agency [47,48] and the EC [49].

In this paper, we employ an indicator-based assessment, usually applied to provide early insights and rapid screening [50]. Our framework draws on previous pivotal applications [34,51–54]. The analysis is inspired by the ESPON climate project framework [12], adapted to meet the terms of the reconciled climate risk terminology. The ESPON Climate change project has developed a comprehensive assessment framework and used it to reveal the variation of the climate change-related challenges across the European regions. A similar approach has been used in the context of Italian climate change adaptation planning. Italy adopted the National Strategy for Climate Change Adaptation (SNAC, Strategia Nazionale di Adattamento ai Cambiamenti Climatici) in 2015 [55]. As a next step, the Italian Climate Adaptation Plan (PNACC, Piano Nazionale di Adattamento ai Cambiamenti Climatici) [56] was elaborated over 2016–2017. In the analysis, we assume that climate change risk arises from the interplay of climate change-related (amplified) hazards, exposure and vulnerability of human and natural systems. Our analysis draws on the modified conceptual framework of the CRI developed for the purpose of PNACC. Whereas PNACC concentrates on medium-long timescales, in this paper we also explore long-term impacts of climate change. PNACC relies on a high-resolution regional climate model developed and successfully tested for Italy [57]. For the purpose of this paper, we employ an ensemble of coarser regional climate simulations obtained from the EURO-CORDEX initiative [58]. By using a multi-models' ensemble, we can explore the effects of uncertainties stemming from model initialization, parametrization and downscaling.

## 2. Data and methods

The main objective of the CRI is to support national authorities in designing adaptation policies and plans, guiding the initial problem formulation phase (identification of key threats, issues and vulnerabilities), as well as a quick screening and prioritization of areas (regions or provinces) with a higher propensity to be adversely affected by climate extremes.

**Figure 1.**
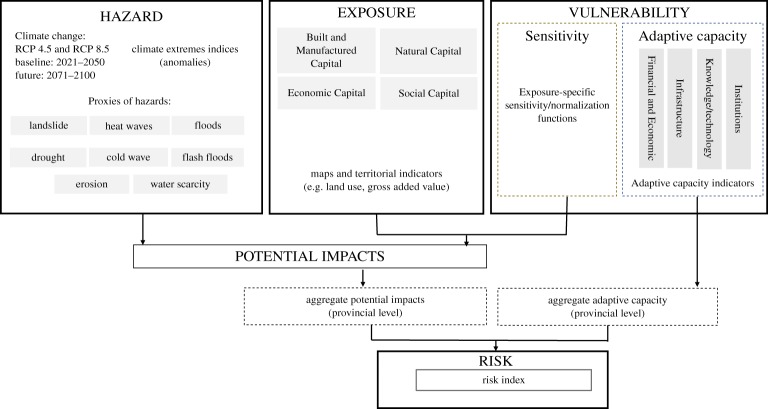
Methodological framework of the CRI (source: adapted from [12]).

The CRI (figure 1) combines (i) climate change-amplified hazards approximated by anomalies of selected extreme climate indices (table 1); (ii) high-resolution indicators of exposure of chosen key economic, social, natural and built- or manufactured capital (MC) assets and (iii) vulnerability which comprises both present sensitivity to climate-induced hazards and AC.

**Table 1.** Description of extreme climate indices (CEI) used in the study.

short name	long name	description	units
R95P	total annual precipitation (PR) from heavy rain days	annual sum of daily PR > 95th percentile	mm
RX1DAY	maximum 1-day PR	maximum amount of daily PR (annual)	mm
R20MM	number of very heavy rain days	annual count of days when PR ≥ 20 mm	days
RJJA	total PR in summer months	sum of daily PR (June–August)	mm
PRCPTOT	annual total wet-day PR	sum of daily PR > = 1.0 mm (annual)	mm
CDD	consecutive dry days	maximum number of consecutive dry days (annual) when PR < 1.0 mm, also referred to as ‘longest dry spell’	days
HWM-TX95P	heat wave magnitude (HWM) as defined by 95th percentile of TX	mean temperature across all individual annual heat waves	°C
CWM-ECF	cold wave magnitude (CWM) as defined by the excess cold factor (ECF)	mean temperature across all individual annual cold waves	°C^2^
SPI-3	3-month Standardized Precipitation Index (SPI), we use only the severe (S) [−1,99; −1,5] and extreme severe (E) [−2,99; – 2,5] drought events	a drought measure specified as a PR deficit on 3-month scale	none
SPI-12	12-month SPI, we use only the severe (S) and extreme severe (E) drought events as in SPI-3	a drought measure specified as a PR deficit on 12-month scale	none

The methodological framework draws on the reconciled concepts of climate risk [59]. In this framework hazard, exposure and vulnerability indicators are selected, normalized and mapped through a tiered aggregation. Vulnerability is captured partly by the sensitivity (or propensity) of the capital assets to be adversely affected by the hazards (e.g. physical and geological characteristics and land cover/use); and partly by the AC, represented by indicators refering to lower-level administrative and statistical units (i.e. the Italian provinces, level 3 in the EU statistical units' nomenclature). Hazard, exposure and sensitivity are first combined in the assessment of potential impacts at 1 × 1 km grid resolution, and subsequently combined with the AC to estimate the final risk index. The AC in our framework denotes the cumulative resources available to respond to or lessen the impact of climate change and is estimated at a provincial level. This is why AC cannot be combined with the sensitivity component of our framework first, and is combined with the potential impacts aggregated at the provincial level.

To assess climate change-related hazards, we use bias-corrected climate simulation from a high-resolution (0.125° grid approx. 12.5 km) ensemble of multi-Regional Climate Models (RCMs), publicly available from the European branch of the Coordinated Downscaling Experiment EURO-CORDEX^1^ [58]. The RCMs used in our study are (i) REMO2009, (ii) RCA4, (iii) HIRHAM5, (iv) CCLM4–8-17, and (v) RACMO22E (hereafter we refer to these models as CCLM, HIRHAM, REMO, RCA, RACMO). Further details of the modelling groups along with their driving GCMs and bias-correction data are provided in the electronic supplementary material, table S1. We focus on two future periods (2021–2050 and 2071–2100), under the Representative Concentration Pathway (RCP) scenario 4.5 [60], relative to the baseline reference period of 1961–1990. By incorporating data from both multiple RCMs and multiple ensemble realizations of individual RCMs (electronic supplementary material, table S1), our analysis accounts for uncertainty stemming from model initialization, parametrization and downscaling. For the assessment of climate change-related hazards, we employ a wide array of climate extreme indices (CEI; table 1) developed by the Expert Team on Sector-specific Climate Indices (ET-SCI) (https://www.wcrp-climate.org/data-etccdi) The CEI are computed at the grid-scale for the implicit spatial and chosen temporal domains by using the simulated daily meteorological variables: (i) maximum near-surface air temperature (TX), (ii) minimum near-surface air temperature (TN) and (iii) near-surface precipitation (PR). The CEI are considered as proxies of the relevant hazards associated with climate extremes such us drought, heat and cold waves, floods, flash floods, landslides, soil erosion and water scarcity. Further details on the methodology of assembling the CEI are provided in the electronic supplementary material.

Exposure and sensitivity assessment is based on indicators representing the degree to which the elements at risk, i.e. manufactured, natural, social and economic capital are exposed to and adversely affected by climate-related hazards. The elements at risk were selected according to a consolidated practice in climate risk and sustainability assessments [61]. MC refers to material goods or fixed assets which support the production process (e.g. industrial machines and buildings); Natural Capital (NC) comprises natural resources and processes (renewable and non-renewable) producing goods and services for well-being; Social Capital (SC) refers to the factors building human capital at the individual (people's health, knowledge, skills) and collective (institutional) level (e.g. families, communities, organizations and schools); finally, Economic Capital (EC) includes owned and traded goods and services. The indicators were chosen from among those commonly used in the literature (see §1). The choice was driven by expert judgements and stakeholder consultations.

The exposure indicators (table 2) refer to socio-economic attributes (e.g. population density (PD) and gross added value), infrastructure and productive areas (e.g. roads and railways, industrial areas), distribution of natural resources (e.g. forests and protected areas) and to the features of the land (e.g. impervious surface or erodible soils). The main sources of data used for MC and NC indicators included COPERNICUS Earth Observation System, the European Soil Data Centre (ESDAC), the European Environmental Agency (EEA), the Organization for Economic Co-operation and Development (OECD) and other open datasets. The indicators used for SC and EC, instead, rely on our previous work. All exposure and sensitivity indicators are represented as a regular square grid with a spatial resolution of 1 × 1 km.

**Table 2.** Exposure and Sensitivity Indicators. Note: OSM, Open Street Map; CLC, CORINE Land Cover 2012; ESDAC, European Soil Data Centre, COPERNICUS (before GMES Global Monitoring for Environment and Security) Earth Observation System.

elements at risk	code	exposure indicators [unit]	source
Manufactured Capital	MC1	Density of infrastructure (roads and railways) [m]	OSM, 2016
	MC2	Urban areas (CLC2012 class 1.1) including high-density build-up areas (1.500–50 000 inhabitants km^−2^, CM2a) and build-up areas (300 inhabitants km^−2^ – 5000 inhabitants km^−2^, CM2b) [m^2^]	COPERNICUS, CLC 2012, EUROSTAT
	MC3	Industrial areas (CLC2012 class 1.2) [m^2^]	CPERNICUS, CLC 2012
	MC1–3	Impervious surfaces (high-resolution (10 m) layer HRL, 2012) [m^2^]	COPERNICUS, ISPRA
Natural Capital	NC1	Forest areas (CLC2012 class 3.1) [m^2^]	COPERNICUS, CLC 2012
	NC2	Natural Protected Areas (NPAs), including NATURA 2000 sites, national and regional protected areas [m^2^]	EEA, 2016
	NC3	Soil erodibility	ESDAC
Social Capital	SC1	PD based on census data (2011, 250 m grid) [inhabitants/km^2^]	Based on own work and described in [62]
	SC2	Structural dependency index	
Economic Capital	EC1	Gross Added Value—agriculture	
	EC2	Gross Added Value—industry	
	EC3	Gross Added Value—services	

For indicators representing the NC and the MC, the grid cell values represent areas (or lengths) of the underlying land cover types (features). Forest cover (NC1) and protected areas (NC2) are based on the CORINE Land Cover 2012 (bit.ly/2EsyBnz), the database of nationally designated protected areas and the Natura 2000 areas (bit.ly/2szAJ7i and bit.ly/2HJ69uH). According to the nomenclature of CORINE land cover (class 3.1), NC1 includes areas occupied by forests and woodlands with a vegetation pattern composed of native or exotic coniferous and/or deciduous trees and able to be used for the production of timber or other forest products. NC2 contains the extension of the Special Protection Areas (SPA) and the Sites of Community Importance (SCIs) under the Natura 2000 Network. Areas more prone to soil erosion (NC3) are based on the elaboration of the ESDAC [63] and include areas with soil loss rates higher than 5 t ha^−1^ yr^−1^. Social and economic exposure data are based on high-resolution gridded demographic and gross added value (GVA) by macroeconomic sectors. The former include PD and the Total Age-Dependency rate (TDR), which expresses the share of dependent (less than 15 and more than 65 years) over non-dependent population [64,65]. Demographic indicators are derived from the latest statistical census (in 2011), transformed onto a 1 × 1 km grid by using a combination of high-resolution land use and soil impervious data [66] as ancillary data. The detailed description of the dataset and the dasymetric mapping algorithm are used to go beyond the scope of this paper and can be found in [67]. Spatial distribution of GVA is based on the subnational economic accounts and derived analysis for the Labour Market Areas (LMA, or local labour systems, SLL). LMA are ‘sub-regional geographical areas where the bulk of the labour force lives and works, and where establishments can find the largest amount of labour force necessary to occupy the offered jobs’ [67]. LMA consists of one or more contiguous municipalities. For each macroeconomic sector, GVA is proportionally split across the respective productive area identified by land cover classes. Namely, GVA density is estimated for each LMA: (i) over the total LMA area, excluding steep slopes and quotas over 600 m; (ii) over the total industrial area, and (iii) over total population for services produced. This method assumes a uniform density of agricultural and industrial GVA within each LMA. GVA related to services is assumed proportional to the PD. Manufactured and built-environment exposure data are derived from land cover and further classified by Eurostat into high-density clusters (HDC), urban clusters (UC) and rural settlements (bit.ly/2EsyBnz). Areas are classified based on geographical contiguity in combination with population size density within each cluster. HDC represent urban areas with at least 1500 inhabitants per square kilometre and a minimum population of 50 000, corresponding to major towns and cities. UC include smaller urban settlements, with a PD of at least 300 inhabitants per square kilometre and a minimum population of 5000. Other smaller, scattered residential areas are part of rural settlements.

Exposure layers were first transformed to a 1 × 1 km grid and successively normalized (transformed into [0,1] range) by using linear (max-min) or nonlinear (sigmoid, S-shaped) functions. The nonlinear normalization was used for demographic indicators (SC1 and SC2 in table 2). See the electronic supplementary material for more information. The anomalies of the extreme climate indices were standardized by using *z*-scores, i.e. divided by standard deviation of the benchmark past period (1960–1990). The layers of standardized climate indices and normalized exposure were spatially overlaid and multiplied, and the intermediate results again normalized. The indicators of built capital were overlaid (multiplied) with anomalies of extreme climate indices R95P, RX1D and R95P; the NC indicators with CDD, R20MM and SPI-3; the social capital indicators with R95P, RX1D, R95P, SPI-12, PREC-TOT, CWM and HWM (for the high-density clusters HDC and urban clusters UC); and economic capital indicators with R95P, SPI-3, SPI-12, RJJA and PREC-TOT. The electronic supplementary material, table S3, shows how the normalized exposure indicators were combined with the standardized hazard indicators.

For the successive aggregation, we use simple weighted addition (SAW) for each RCM separately, and the ordered weighted average (OWA) [68]. SAW uses equal weights for intermediate indices. OWA applies three sets of weights, OWA MAX [1/3, 1/3, 1/3, 0, 0], OWA MIN [0, 0, 1/3, 1/3, 1/3] and OWA MED [0, 1/3, 1/3, 1/3, 0]. OWA MAX is the average of the RCMs at the higher end of the range of projected climate hazards, hence it can be associated with a risk-averse attitude of policy makers. The OWA MIN places more emphasis on the lower end of the projected hazard range. OWA MED comes closest to an arithmetic average across the RCMs, with the difference that it averages all but the RCMs with the lowest and the highest value of the standardized anomalies. The aggregation methods are further discussed in §3. We acknowledge that the choice of weighting method encompasses a certain degree of subjectivity. Different weighting and aggregation methods allow for a different degree of compensation, which should reflect the preferences of decision or policy makers and the purpose for which the assessment is conducted. In our analysis, we concentrate on identifying the whole range of possible CRI values.

For ACI, we used the analysis described in depth in [69]. It combines a set of 10 indicators related to available economic resources (such as provincial GDP, at-risk-of-poverty rate and unemployment), infrastructure, knowledge and technology (e.g. Internet access, educational level and patent applications), and institutional quality (based on the institutional quality index as in [70]). The methodology used for the ACI index goes beyond the scope of this paper, and the ACI scores are included in electronic supplementary material, table S3, for comparison only. Further information about the ACI can be found in the electronic supplementary material.

## 3. Results

**Figure 2.**
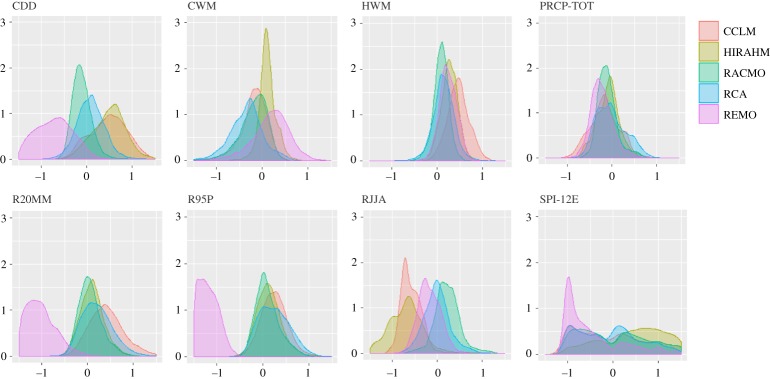
Density distributions of extreme climate indices (abbreviations as in table 1) for period 2021–2050.

**Figure 3.**
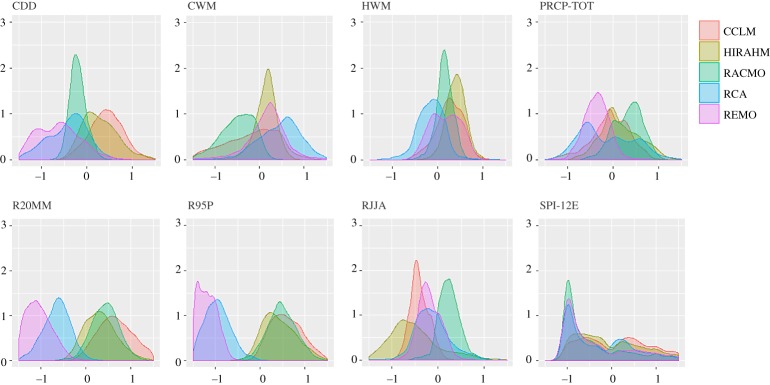
Density distributions of extreme climate indices (abbreviations as in table 1) for period 2021–2050.

The anomalies of climate indices differ across RCMs in sign, magnitude and spatial distribution of the projected change, both within and across the future periods (2021–2050 and 2071–2100). The density distributions in figures 2 and 3 allow for comparing the expected changes across models and time periods. Some indices (e.g. PREC-TOT and HWM in figure 2 display similarly shaped distributions with slightly different means. The distribution of other indices differs both in shape and mean values. For example, REMO model projects an opposite change compared to CCLM and HIRAHM for CDD index, and differs considerably from all other models in R20MM and R95P. Figure 3 shows how these differences are further amplified in 2071–2100.

Rank correlations (electronic supplementary material, figures S1 and S2) express the degree of similarity between two rankings, i.e. the extent to which, if one index increases, the other indices tend to increase as well. The negative correlations indicate the discordance in the direction of the projected change but not necessarily in its sign. For example, REMO and CCLM are fairly correlated, although generally pinpointing opposite direction of the change. Low positive correlations indicate discordance in the magnitude of change. The electronic supplementary material, figures S1 and S2, shows that low yet significant rank correlations across the different RCMs are a norm, and this embodies uncertainty in future climate projections that we take into account by using an ensemble of climate models.

**Figure 4.**
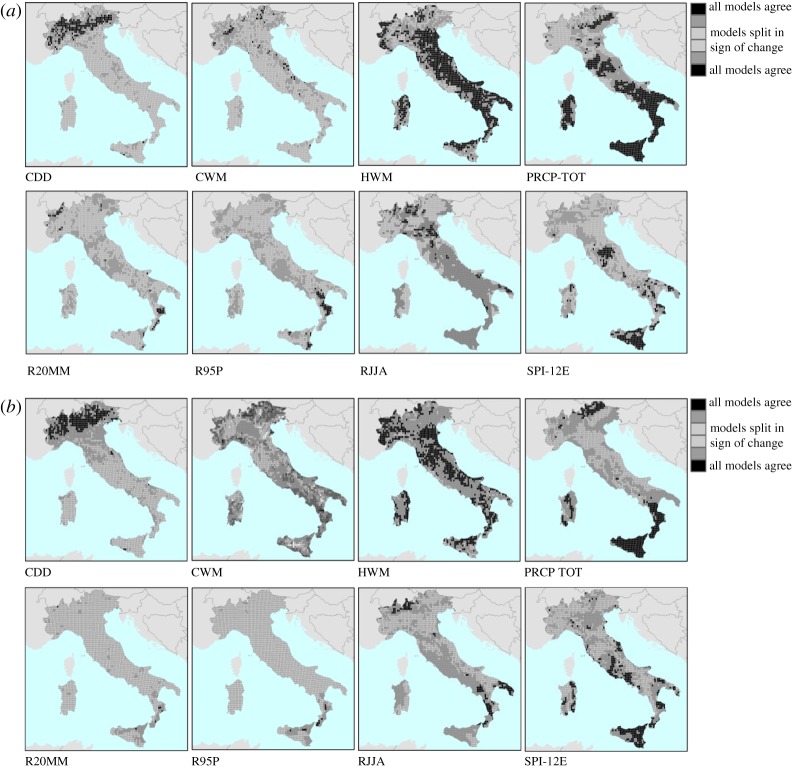
(*a*) Concordance in the anomalies of climate extreme indices across the various RCMs for 2021–2050. (*b*) Concordance in the anomalies of climate extreme indices across the various RCMs for 2071–2100. (Online version in colour.)

Figure 4 shows the spatial distribution of the models' convergence, that is the locations in Italy of areas for which the models agree on the sign of the change, either negative or positive. What emerges is a recurrent spatial pattern in the climate projections: HWM shows a high concordance across the whole country; and a regionally constrained convergence in the period 2021–2100 can be noticed for CDD (in the Alps) and PRECTOT (in southern and a large part of central Italy). The simulations are consistent on SPI-12E for Sicily, while characterized by large heterogeneity for other parts of the country. Note that the SPI-12E indicator is characterized by a weak overall agreement across RCM, nonetheless, convergence can be found for some areas. In the period 2071–2100 in most cases the initially observed convergence is partially eroded and the difference across the RCMs is generally amplified.

Understanding the differences encountered in the climate extreme indices is central to the next step of our analysis, in which the climate indices are analysed in areas where extreme phenomena are expected to produce the largest impacts. The index-based approach combines present-day exposure and sensitivity with the climate indices, using the former to restrict the focus or as weights of areas of particular concerns. We combine standardized anomalies of the extreme PR and temperature indices with the elements embedding exposure and sensitivity to the climate change-induced alteration of extreme climate phenomena.

We employ two aggregation techniques. In the first, the index of potential climate change impacts (CPI) is estimated for each RCM separately, as described in §2 for a 1 × 1 km regular square grid and successively summarized at the provincial (NUTS3) level. For each province, we thenceforth determine the minimum, maximum and median values of the standardized index from across the range of the five RCM models. For each province the min, max and median values can originate from assessments driven by different RCMs and denote the low, high and medium levels of the expected alteration of climate risk. In the second aggregation method, we employ the OWA (ordered weighting average) operator. OWA entails a parametrized class of aggregation operators which comprise also ordinary min, max and median descriptors. OWA operator is nonlinear, bounded, monotonic and symmetric [68]. OWA uses a set of arbitrary chosen weights that are applied not to the scores obtained for each RCM but thereby to rank positions in the order from the largest one to the lowest one. We use three different OWA operators corresponding to OWA MAX, OWA MIN and OWA AVE. OWA MAX applies a set of weights (all equal 0.33) to the uppermost ranked scores (that is those at rank positions 1–3), OWA MIN applies the same set of weights to the lowest ranked scores (at rank positions 3–5), and OWA AVE applies the vector of weights to the middle ranks (positions 2–4). CPI OWA scores are determined as follows: (i) we first estimate OWA scores for each extreme climate index separately before (ii) these are combined with the indicators of the exposure sensitivity. In doing so, the aggregation is not performed for each RCM separately but by using the OWA aggregated extreme climate indices. The choice of aggregation methods is to some extent arbitrary. By using SAW, we assume that all climate change-amplified hazards addressed in this analysis (e.g. floods, droughts, landslides, water scarcity/security, extreme temperatures—heat and cold waves, and soil erosion) are equally important within the frame of the CRI. The scope of our analysis is to explore the uncertainty stemming from different climate projections. We estimate a range of different rank positions for each administrative district (province) based on ensemble of RCMs. This range would be similar if different weighting and aggregation methods were applied. By using OWA, on the other hand, we explore to what extent the ensemble mean (OWA AVE) or endpoints (OWA MIN and OWA MAX) reproduce or contract the range estimated by SAW.

**Figure 5.**
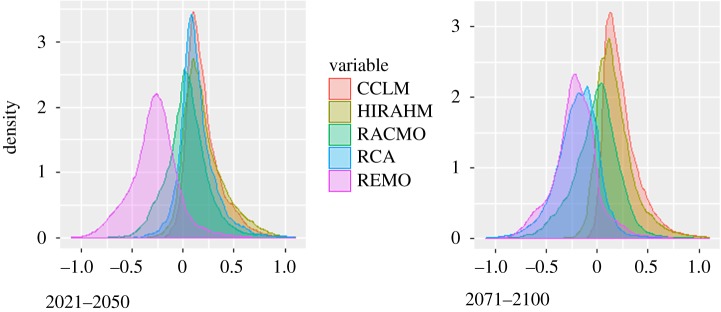
Density distribution of the CPI scores by different RCMs.

**Figure 6.**
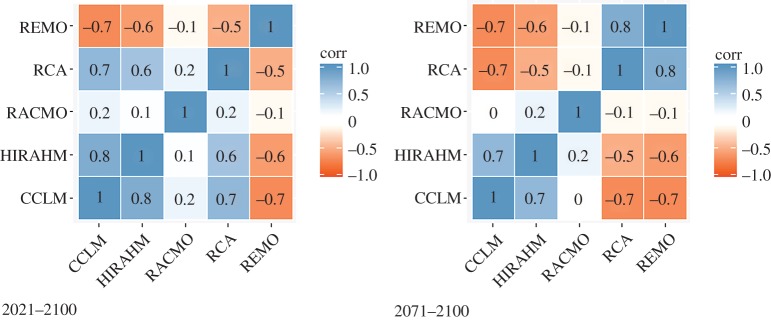
Rank correlations between the CPI scores by different RCMs.

**Figure 7.**
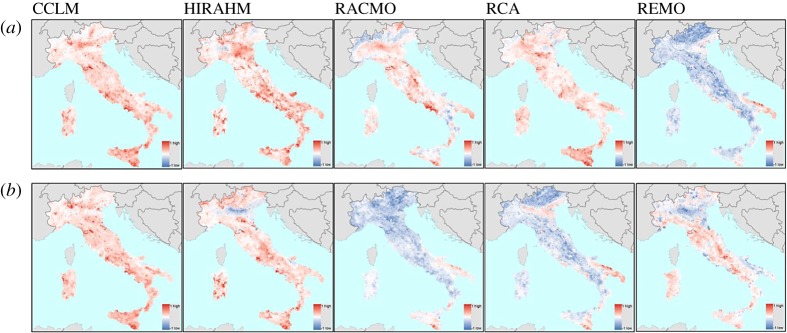
Aggregate CPI by RCMs and periods, (*a*) 2021–2050 and (*b*) 2071–2100.

Figures 5–7 show the rank correlations, density distribution and spatial distribution of the CPI scores obtained by using the first (crisp) aggregation method. The results are consistent with the differences observed in the CEIs. For the period 2021–2050, the distributions of the CPI scores across all RCMs except REMO display similar symmetrical shapes and mean values, while differing in dispersion. The distribution of the scores obtained by using the REMO model is also symmetrical but its mean value is shifted to the left from the other RCMs. CPI driven by REMO model is negatively correlated with all other models, particularly with CCLM and HRAHM. For the period 2071–2100, the distributions of the scores obtained for RACMO and RCA models becomes more dispersed (stretched), with mean value much closer to the REMO model. Other model distributions remain almost unchanged. In this period, both REMO and RCA are negatively correlated with CCLM and HIRAHM. Figure 7 shows the spatial pattern of the CPI scores, panel (*a*) for the period 2021–2050 and panel (*b*) for the period 2071–2100. The negative values of the CPI scores stand for less pronounced climate extremes under the conditions of future climate change.

**Figure 8.**
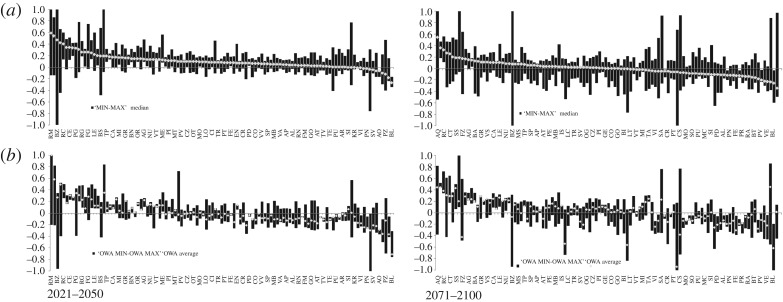
Comparison of the aggregate results by provinces (NUTS3) and period of reference. (*a*) The results of CPI by provinces, ordered by the CPI median values; (*b*) the CPI-OWA index, reflecting the ranking in the respective panel (*a*).

**Figure 9.**
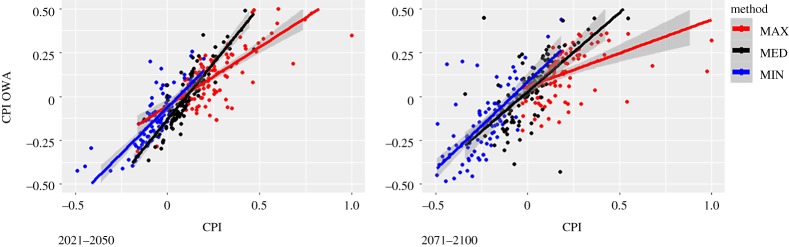
Comparison of the aggregate results by aggregation method and period of reference.

**Figure 10.**
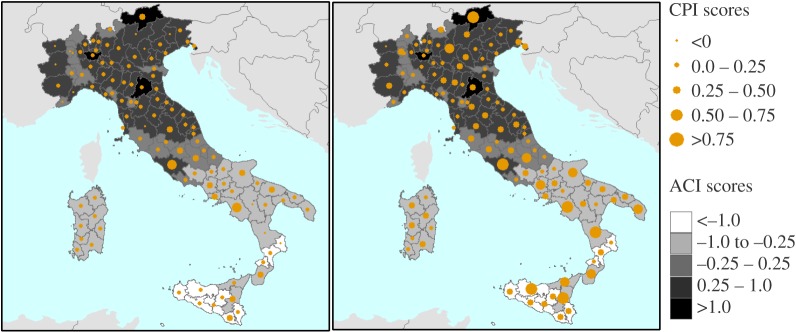
Aggregate results CRI MED (left) and CRI MAX (right) for 2021–2050.

Figure 8 shows the CPI (*a*) and CPI OWA scores (*b*) aggregated by provinces. The figures portray both the full range of expected anomalies of extreme climate phenomena (represented as floating columns) and the median or average value (represented by points). The figure displays a rather low range of the CPI scores for all but a few provinces for the period 2021–2050. By the end of the century, the ranges broaden up. The OWA aggregation operators (figure 8*a*) do not substantially reduce the range (uncertainty) and for at least some cases the ranges are further amplified. The comparison between CPI and CPI OWA scores are further explored in figure 9, which also shows the fitted regression lines for each aggregation operator separately. The figure shows that OWA generally attenuates the aggregate index scores, and this mitigating effect is largest for the MAX operator, whereas the MED (average or median) operator yields almost equivalent scores both for CPI and CPI OWA. Using ensemble mean of RCMs would produce a similar, or even more pronounced effect. This is why computing an aggregate index for each RCM separately is a better choice to convey the full range of uncertainty in the regional climate simulations, even if one then chose to apply one or more aggregation operators to determine the final ranking. Figure 10 shows the CPI MED and CPI MAX for 2021–2050, along with the standardized scores of the climate adaptive index (ACI). The latter shows a higher performance of the northern and central provinces compared with the southern ones. The correlation between the CPI and ACI scores is significant but rather low (0.4) which indicates a mixed pattern of climate risk and AC. Table S3 in the electronic supplementary material contains the results for all aggregation methods and both periods of analysis.

## 4. Discussion and conclusion

An index-based climate risk assessment is a proxy substitute of an in-depth assessment that should ideally be carried out for each climate change-related hazard, identifying the expected damage or loss. Index-based assessment is useful when hazard-specific damage functions are not available or easily transferable to a specific application context. In this paper, we describe an index-based methodological approach to assess climate change risk in Italy. This extends the approach used within the framework of the Italian climate change adaption planning by exploiting an ensemble of bias-corrected RCM simulations made available by the EURO-CORDEX initiative.

The index explores the changing climatic conditions in Italy and the associated risks stemming from extreme climate events. It is intended to complement the existing knowledge and provide a viable alternative for the identification of adaptation priorities, until more sophisticated and data-intensive risk assessments are performed. The conceptual framework underpinning the risk index is less accurate than process-based numerical models, and is therefore designed for meso-scale applications that are relevant for national (or supra-national) planning. As such, even though it makes it possible to rank subnational territorial units, it is not suitable to guide the identification and implementation of specific adaptation measures at the local scales that need a much higher data resolution and richness.

Our analysis has highlighted a sizeable discordance in the projected extreme climate indices produced by the state-of-the-art RCMs. This variability should be fully explored and exploited, rather than neglected. Firstly, and most obviously, to indicate where uncertainties are and where to invest in research; secondly, on which variables most robust decisions could/should be made. With respect to this, we have shown that the divergence between the model simulations may be large enough to limit the use of the ensemble mean. In these cases, while the mean can be useful as a benchmark, the policy and decision makers may be well advised to explore the full range of possible climate alterations, and identify ways that turn the uncertainties into well pondered and designed choices.

Notwithstanding the model differences, at least partly-consistent patterns in climate hazards can be found and used as a starting point for adaptation planning. This is true especially for water availability decline and increased incidence of extreme drought episodes in the south. Yet, our analysis has challenged the stereotype view of the south–north divide, rooted in persisting economic and social inequalities. We have shown that the north is neither insulated form substantial climate change impact nor better prepared to cope with them. On the contrary, parts of the southern provinces may find it rewarding to boost their resilience and capacity to adapt to climate change.

Eventually, the results of our analysis make possible sub-national climate adaptation planning and needs assessment. The methodology employed is flexible enough to produce the climate-risk-related ranking of subnational administrative and statistical units. The confrontation of the potential climate change impacts and AC can serve as a yardstick for priority planning and the initial allocation of funds for more in-depth climate risk assessment. Sub-national units facing high risk and low AC should be encouraged, by public policy-making bodies or private entities and community organizations. At the regional (NUTS2) level, our methodology offers innovation that may be harnessed for the purposes of updating the ESPON 2011 climate change assessment and informing EU Cohesion policy. We estimate a range of different rank positions for each administrative district (province) based on the ensemble of RCMs. By using OWA, we explore to what extent the ensemble mean or endpoints reproduce or contract the range estimated by SAW. To facilitate the use of CRI by lower administrations, we are developing a web-based platform which makes possible an easy access to and handling of a variety of hazard, exposure and vulnerability indicators, so that decision and policy-makers can customize the index for their specific purposes.

There are several areas in which our methodology can be further improved or tailor-made for specific decision-making contexts. First, our analysis is limited to climate extremes related to temperature and PR, but can be applied to other hazards. The choice of the indices can be adapted to specific policy needs. We have used daily aggregated data which are unable to discern hazard risk at a sub-daily temporal scale such as maximum hourly PR. This can be amended. Similarly, and despite data constraints particularly relevant for socio-economic indicators, additional indicators for both exposure and AC could be included to account for regional specificities and peculiarities. Projection of exposure (e.g. PD, wealth and land cover) and AC can lead, at least to some extent, to different results, at the cost of larger uncertainty. And a more accurate account of susceptibility to harm based on projected changes in intensity and frequency of climate-related hazards (e.g. by using fragility curves) and as a result of public and private investments into disaster risk reduction can yield better results.

Furthermore, it is possible to connect methodologies like the one we used here as an input for economic models at a subnational (regional or provincial) scale to conduct a full-fledged economic impact assessment. This normally requires assessment of climate-change-related hazards and risk in terms of capital asset losses, but the high-resolution exposure data we have used for the analysis is amendable for this type of analysis. It is possible, for example, to estimate production losses based on physical damage to asset and business interruptions, but for this purpose, the impact and damage functions have yet to be developed or improved.

## Supplementary Material

Additional figures and tables

## Supplementary Material

Table 3 - final results by provinces

## Footnotes

1 Data were accessed last on 15 March 2017 from the Earth System Grid Federation (ESGF) node https://esgf-data.dkrz.de/search/cordex-dkrz/

## Data accessibility

The data presented in this paper are part of the electronic supplementary material and after the review will be uploaded in the open data scientific database with a DOI number.
