# Supplementary material for: Climate risk index for Italy
Source: Philos Trans A Math Phys Eng Sci. 2018 Apr 30;376(2121):20170305. doi: 10.1098/rsta.2017.0305 (PMC5938637; doi:10.1098/rsta.2017.0305)
Supplement: Additional figures and tables [file rsta20170305supp1.docx]

**Supplementary material**

# Introduction

We describe a climate risk index that has been developed to inform national climate adaptation planning in Italy and that is further elaborated in this paper. The index guides the initial problem formulation and helps to identify administrative areas with higher propensity to being adversely affected by climate change. The key components of the climate risk index are: hazard (H), i.e. the potential occurrence of a damaging physical event related to climate trends or climate extremes; exposure (E), i.e. the presence of environmental, social or economic elements in places that could be adversely affected; and vulnerability (V), i.e. the predisposition to be adversely affected, comprising a variety of concepts including sensitivity or susceptibility to harm and the lack of capacity to cope and adapt (1).

***Comparison of conceptual frameworks of risk and vulnerability***

The concepts of risk, hazard and vulnerability have been interpreted in different ways over time, reflecting the evolution in a variety of scientific disciplines related to climate change adaptation and disaster risk reduction (2–4). Our methodological framework draws on the terminology of the Intergovernmental Panel on Climate Change (IPCC). Table 1 shows how IPCC’s definitions evolved over time.

|  | **Climate Risk Index (CRI)** | **IPCC-AR4 (2007), IPCC-AR5 (2014) and ESPON (2011)** |
| --- | --- | --- |
| **HAZARD** | **IPCC-AR5** | **IPCC-AR5:** The potential occurrence of a natural or human-induced physical event or trend or physical impact that may cause loss of life, injury, or other health impacts, as well as damage and loss to property, infrastructure, livelihoods, service provision, ecosystems and environmental resources. In this report, the term hazard usually refers to climate-related physical events or trends or their physical impacts |
| **EXPOSURE** | **IPCC-AR5** | **IPCC-AR4 and ESPON:** The nature and degree to which a system is exposed to significant climatic variation.  **IPCC-AR5:** The presence of people, livelihoods, species or ecosystems, environmental functions, services, and resources, infrastructure, or economic, social, or cultural assets in places and settings that could be adversely affected. |
| **VULNERABILITY** | **IPCC-AR5** | **IPCC-AR4 and ESPON:** The degree to which a system is susceptible to, and unable to cope with, adverse effects of climate change, including climate variability and extremes. Vulnerability is a function of the character, magnitude, and rate of climate variation to which a system is exposed, its sensitivity, and its adaptive capacity.  **IPCC-AR5:** The propensity or predisposition to be adversely affected. Vulnerability encompasses a variety of concepts and elements including sensitivity or susceptibility to harm and lack of capacity to cope and adapt |
| **RISK** | **IPCC-AR5** | **IPCC-AR5:** The potential for consequences where something of value is at stake and where the outcome is uncertain, recognizing the diversity of values. Risk is often represented as probability or likelihood of occurrence of hazardous events or trends multiplied by the impacts if these events or trends occur. In this report, the term risk is often used to refer to the potential, when the outcome is uncertain, for adverse consequences on lives, livelihoods, health. |
| **SENSITIVITY** | **IPCC AR4 and ESPON** | **IPCC-AR4 and ESPON:** The degree to which a system is affected, either adversely or beneficially, by climate variability or change. |
| **ADAPTING CAPACITY** | **IPCC-AR5** | **IPCC-AR4:** The ability of a system to adjust to climate change (including climate variability and extremes) to moderate potential damages, to take advantage of opportunities, or to cope with the consequences.  **ESPON:** The ability of a natural or human system to adjust to climate change (including climate variability and extremes) to moderate potential damages, to take advantage of opportunities, or to cope with the consequences.  **IPCC-AR5:** The ability of systems, institutions, humans and other organisms to adjust to potential damage, to take advantage of opportunities, or to respond to consequences. |
| **POTENTIAL IMPACTS** | **IPCC AR4** | **IPCC AR4:** all impacts that may occur given a projected change in climate, without considering adaptation. |

**Supplementary Table S1:** Comparison of methodological frameworks for vulnerability and risk.

The IPCC Special Report on Managing the Risks of Extreme Events and Disasters to Advance Climate Change (SREX) (5) and the Fifth Assessment Report (AR5) moved toward a risk-centred framework (1,6) in which risk is a function of hazard, exposure, and vulnerability. The framework used for previous IPCC Assessment Reports put emphasis on vulnerability (7–10), denoted as a function of exposure, sensitivity and adaptive capacity (11–15).

Our analysis is inspired by the ESPON Climate Change assessment framework (10), adapted to the reconciled climate risk terminology. ESPON developed a comprehensive framework to assess the climate change-related challenges across Europe. In that framework, exposure and sensitivity are combined to assess the potential impacts of climatic change. Exposure analysis is based on CCLM climate model, using IPCC A1B scenario (1961-1990 and 2071-2100) for each of the exposure indicators (e.g. change in annual mean temperature, relative change in annual mean precipitation in summer months, etc.). To assess the sensitivity, ESPON defines five (physical, social, environmental, economic and cultural) dimensions which indicate how the overall system is affected by climatic changes. Exposure and sensitivity were subsequently aggregated, and combined with adaptive capacity.

In our framework, we combine hazards and exposure to evaluate the potential climate change-related impacts. We use standardised anomalies of selected extreme climate indices derived from high resolution regional climate models as proxies of climate change-altered weather and climate-related hazards. Exposure elements have been assessed through indicators reflecting socioeconomic attributes, infrastructure and productive areas, distribution of natural resources and land features. To normalize the indicators we use min-max and S-shaped functions. The exposure/sensitivity indicators were multiplied by hazard indicators and then aggregated by means of simple additive weighting (SAW) and ordered weighted average (OWA). The aggregated potential climate impacts (CPI) are combined with adaptive capacity index (ACI) to determine climate risk index (CRI).

*The National Strategy and Plan for Climate Change Adaptation in Italy.*

Climate change risk index was developed to inform the climate adaptation planning in Italy. The Italian Climate Adaptation Plan (PNACC - *Piano Nazionale di Adattamento ai Cambiamenti Climatici*) (MATTM, 2017) refined the assessment of climate change related risks and elaborated on the adaptation actions identified in the Italian National Strategy for Climate Change Adaptation (SNAC - *Strategia Nazionale di Adattamento ai Cambiamenti Climatici*) (17), adopted with the [Ministerial Decree](http://context.reverso.net/translation/english-italian/Directorial+Decree) No 86 of June 16th, 2015. SNAC consists of (i) a review assessment of climate change impacts and vulnerabilities (18); (ii) a critical review of the European and national legislation related to climate adaptation (19), and (iii) keystones of a National Adaptation Strategy laying down the strategic objectives and main adaptation actions (20). The purpose of the climate change risk index in the PNACC was to identify which areas are more prone to the adverse effects associated with the human-induced climate change in Italy.

# Data and methods

To assess the climate change-related hazards, we use selected climate extreme indices (CEI) from among those developed by the Expert Team on Sector-specific Climate Indices (ET-SCI)^^[[1]](#footnote-1)^^. The climate extreme indices were computed utilizing the R-software package ClimPACT2^[[2]](#footnote-2)^. It requires the three main meteorological variables (tmax, tmin and precip) at daily time steps. We use bias-corrected climate simulation from a high-resolution (0.125° grid ~12.5 km) ensemble of multi-Regional Climate Models (RCMs) from EURO-CORDEX^^[[3]](#footnote-3)^^ (21). The input variables are on a native curvilinear rotated lon-lat grid, which is not directly compatible with the ClimPACT2 package. We therefore employ a series of pre-processing steps using the command line utilities Climate Data Operators (CDO) and netCDF Operators (NCO)^^[[4]](#footnote-4)^^, to not only convert (interpolate) the input netCDF files grid to regular lon-lat at the equivalent native resolution (~0.11°), but also to compute the RCM specific ensemble mean for each variable before computing the CEI. Figures 1 and 2 show the Spearman correlation between the extreme climate indices anomalies. Table S2 provides an overview of the climate models selected to calculate the indices.

Extremes climate indices (**R95p**, **R20**, **rx1d**, **WP**, **SP**, **CDD**, **SPI3**, **SPI12**, **HWM**, **CWA** – for definitions see Table 1 in the manuscript) are used to characterize the natural hazards. Additional information has been obtained from third parties. The areas prone to medium flood hazard (**P2**) and developed for the purpose of the EU Floods Directive were obtained from the Italian Environmental Agency (ISPRA, http://bit.ly/2GhTU7V). The Flash Flood Precipitation Index (**FFPI**) was calculated based on soil type, slope, land cover and water retention capacity. Data on soil profile and water retention capacity was derived from the soil macro-categories produced by the European Soil Data Centre (ESDAC, esdac.jrc.ec.europa.eu, European Commission, Joint Research Centre). The slope was obtained from the digital model (DEM, http://bit.ly/2HgSXxX). The land cover was obtained from Corine Land Cover and soil sealing data (http://bit.ly/2Eo3s4x) of the Copernicus Land Monitoring Service (https://land.copernicus.eu) and ISPRA.

The exposure and sensitivity assessment is based on indicators of manufactured, natural, social and economic capital assets exposed to and adversely affected by climate-related hazards (Table 2 in the manuscript). The Manufactured Capital (MC) refers to material goods or fixed assets which support the production process (e.g. industrial machines and buildings); the Natural Capital (NC) comprises natural resources and processes (renewable and non-renewable) producing goods and services for the well-being; the Social Capital (SC) addressed factors at individual (people's health, knowledge, skills) and collective (institutional) level (e.g. families, communities, organizations, schools); and the Economic Capital (EC) includes owned and traded goods and services.

Urban clusters data was acquired from Eurostat as high-density clusters (*CM2a^[[5]](#footnote-5)^*, urban areas at least 50,000 inhabitants with a density over 1,500 residents/km^2^, http://bit.ly/2Br8LNX) and urban clusters (*CM2b*, urban areas at least 5,000 inhabitants with a density over 300 residents/km^2^, http://bit.ly/2BXPeWr). The soil sealing indicator (*CM1-3*) were obtained from the National Land Consumption Map at 10 meters resolution (2017 edition, v1.0, http://bit.ly/2Eo3s4x). Infrastructures density (*CM1*) was calculated starting from the data related to the road infrastructures, acquired by OpenStreetMap (OSM, http://bit.ly/2EGkjim). Industrial areas (*CM3*) and forest areas (*CN1*) are derived from CORINE Land Cover (http://bit.ly/2EsyBnz). Protected areas (*CN2*) were identified from the inventory of the European Environment Agency (EEA, http://bit.ly/2EwRdCQ) and integrated with the areas of the Natura2000 network (https://bit.ly/2qnqU9g). The soil erosion potential (*CN3*) is calculated based on the “soil erosion by water” index (ESDAC) (22). The indicators SC1-2 and EC1-3 were derived from the high-resolution dasymetric grid developed by the authors (23) for Italy and comprising population, gross domestic value (GDP), land use and cadastral information. The dasymetric mapping consists of overlaying high-resolution population data (at census tracks) and ancillary data. Population density (*CU1*) and the structural dependency index (*CU2*) were calculated based on the 2010 population census for each census section. GDP was derived from the Italian Statistical Office (ISTAT) for the main economic branches at the resolution of the labor market areas (LMA). GDP density was determined in relation to its corresponding production area for each macro-sector. See for a detailed description of the dasymetric methods used.

We employed two aggregation techniques. [1] Using simple additive weighting (SAW), we first calculate the an potential climate change index (CPI) at 1x1km grid resolution for each climate model separately. Subsequently we summed up the grid values within each province (NUTS3 regions). These values were again standardized. Then we estimated min, max and median values, for each province separately, across the climate models. [2] Following the OWA aggregation we first apply the OWA weights to individual proxy hazard indicators and then determine the CPI value, first for 1x1km grid ad subsequently for the provinces. The different aggregation methods yield different values of the CPI index. Using the aggregation above we determine, for each province, the range of possible values, and rank positions, as shown in Figure 8 of the manuscript.

The different aggregation operators entails different degrees of compensation. Therefore the choice of the aggregation operator should reflect the purpose of the assessment and the decision makers’ preferences. Mean aggregation operators are often used to allow moderate level of compensation. Aggregation operators such as quasi-arithmetic mean (generalized mean) and OWA have the ability to control the level of compensation which would be desirable instrument for decision makers. OWA operator provides a family of aggregation operators including many of the well-known operators such as the maximum, the minimum, the k-order statistics (used in the study) and the arithmetic mean. Detailed description of OWA operators can be found in (24,25). The OWA weights we used in our analysis approximate the ensemble means and end points of the range. Table S4 shows the algorithms used to determine the potential climate change impact index (CPI). CPI is constructed by summing up all sub-indices in Table S4.

The adaptive capacity index (ACI) was built around four dimensions and ten indicators at NUTS3 statistical level based on conceptual framework proposed by ESPON (26). The main determinants of the adaptive capacity are economic resources, knowledge and technology, infrastructure and institutions. The indicators for each determinant of the framework have been chosen based on expert judgements and literature review and tested by means of multivariate analysis and principal component analysis. The data obtained from multiple sources. It includes data extracted from the database of territorial indicators for the development policies (27) developed by Italian National Statistical Office (ISTAT) as a part of the sectoral territorial statistical information on structural policies 2010-2014. Additional data was obtained from (28–30). Institutional Quality Index (IQI) (31) was used as proxy for the component related to institutional quality. Indicators were normalized using z-scores standardization. Three sets of weights were applied in the analysis. For a preliminary screening, equal weights were used. In the next step, the same set of weight as in ESPON (2011) was employed which were adapted to the assessment design. The last set of weights was estimated by using principal component analysis which is based on highest possible variability in the indicator set. A detailed explanation of the applied method can be found in (32). SAW was employed to determine the final performance and rankings.

| **RCP** | **RCM (-version no.)** | **RCM Host Institution and country** | **Driving GCM from CMIP5** | **Number of Ensembles^#^** | **Bias-correction method^*^** | **Contact person** |
| --- | --- | --- | --- | --- | --- | --- |
| 2.6 | REMO2009-v1 | Max Planck Institute for Meteorology - Climate Services Centre (MPI-CSC), | MPI-M-MPI-ESM-LR | 2 | **SMHI-DBS45**-*MESAN-1989-2010* | Daniela Jacob ([daniela.jacob@hzg.de](mailto:daniela.jacob@hzg.de)) |
| 4.5 | CCLM4-8-17-v1 | Brandenburg University of Technology (BTU), Germany | CNRM-CERFACS-CNRM-CM5 | 2 | **METNO-QMAP**-*MESAN-1989-2010,* **SMHI-DBS45**-*MESAN-1989-2010* | Klaus Keuler ([keuler@tu-cottbus.de](mailto:keuler@tu-cottbus.de)) |
|  |  |  | ICHEC-EC-EARTH | 2 |  |  |
|  |  |  | MPI-M-MPI-ESM-LR | 2 |  |  |
|  | HIRHAM5-v1 | Danish Meteorological Institute (DMI), Denmark | ICHEC-EC-EARTH | 2 | **METNO-QMAP**-*MESAN-1989-2010,* **SMHI-DBS45***-MESAN-1989-2010* | Jens H. Christensen ([jhc@dmi.dk](mailto:jhc@dmi.dk)) |
|  | RACMO22E-v1 | Royal Netherlands Meteorological Institute (KNMI), Netherlands | ICHEC-EC-EARTH | 2 | **METNO-QMAP**-*MESAN-1989-2010,* **SMHI-DBS45**-*MESAN-1989-2010* | Erik van Meijgaard ([vanmeijg@knmi.nl](mailto:vanmeijg@knmi.nl)) |
|  | RCA4-v1 | Swedish Meteorological and Hydrological Institute (SMHI), Sweden | CNRM-CERFACS-CNRM-CM5 | 1 | **METNO-QMAP**-*MESAN-1989-2010* | Grigory Nikulin ([grigory.nikulin@smhi.se](mailto:grigory.nikulin@smhi.se)) |
|  |  |  | ICHEC-EC-EARTH | 1 |  |  |
|  |  |  | IPSL-IPSL-CM5A-MR | 1 |  |  |
|  | REMO2009-v1 | MPI-CSC, Germany | MPI-M-MPI-ESM-LR | 2 | **SMHI-DBS45**-*MESAN-1989-2010* | Daniela Jacob ([daniela.jacob@hzg.de](mailto:daniela.jacob@hzg.de)) |
| 8.5 | CCLM4-8-17-v1 | BTU, Germany | CNRM-CERFACS-CNRM-CM5 | 1 | **SMHI-DBS45**-*MESAN-1989-2010* | Klaus Keuler ([keuler@tu-cottbus.de](mailto:keuler@tu-cottbus.de)) |
|  |  |  | ICHEC-EC-EARTH | 1 |  |  |
|  |  |  | MPI-M-MPI-ESM-LR | 1 |  |  |
|  | HIRHAM5-v1 | DMI, Denmark | ICHEC-EC-EARTH | 1 | **SMHI-DBS45***-MESAN-1989-2010* | Jens H. Christensen ([jhc@dmi.dk](mailto:jhc@dmi.dk)) |
|  | RACMO22E-v1 | KNMI, Netherlands | ICHEC-EC-EARTH | 1 | **SMHI-DBS45**-*MESAN-1989-2010* | Erik van Meijgaard ([vanmeijg@knmi.nl](mailto:vanmeijg@knmi.nl)) |
|  | REMO2009-v1 | MPI-CSC, Germany | MPI-M-MPI-ESM-LR | 2 | **SMHI-DBS45**-*MESAN-1989-2010* | Daniela Jacob ([daniela.jacob@hzg.de](mailto:daniela.jacob@hzg.de)) |

**Supplementary Table S2:** Details of simulations available and used for computing extreme indices (EURO-CORDEX).

^#^Refers to the total number of ensembles (combination of the driving GCM’s realizations and bias-correction method).

^*^ Bias-correction method (in bold). Reference observational dataset and the period used for the bias correction (in Italics)*.*

| **RCMs 2021-2050** | CCLM | HIRAHM | RACMO | RCA | REMO |  |  |  |  |
| --- | --- | --- | --- | --- | --- | --- | --- | --- | --- |
| REMO | 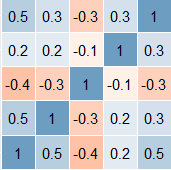 | | | | | 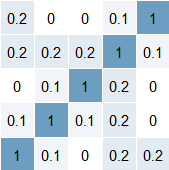 | 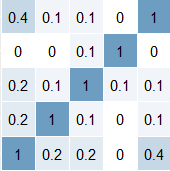 | 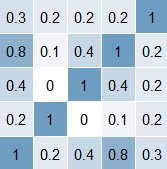 | 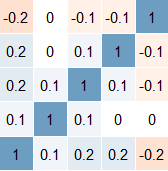 |
| RCA |  |  |  |  |  |  |  |  |  |
| RACMO |  |  |  |  |  |  |  |  |  |
| HIRAHM |  |  |  |  |  |  |  |  |  |
| CCLM |  |  |  |  |  |  |  |  |  |
|  | **CDD** | | | | | **CWM** | **HWM** | **PRCP TOT** | **R20mm** |
| 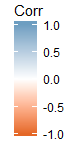 | 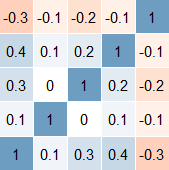 | | | | | 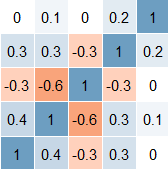 | 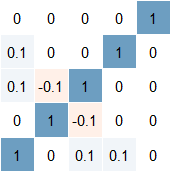 | 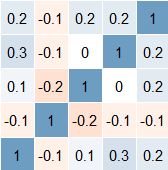 | 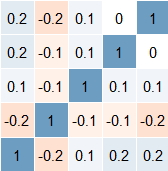 |
|  | **R95p** | | | | | **rJJA** | **rx1day** | **SPI-12E** | **SPI-3E** |

**Supplementary Figure 1:** Spearman pairwise correlations between the standardised anomalies of selected extreme climate indices (abbreviations as in table 1 in the manuscript) for period 2021-2050, significant at 95% confidence level.

| **RCMs**  **2071-2100** | CCLM | HIRAHM | RACMO | RCA | REMO |  |  |  |  |
| --- | --- | --- | --- | --- | --- | --- | --- | --- | --- |
| REMO | 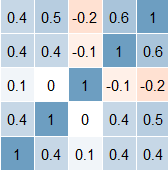 | | | | | 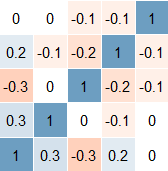 | 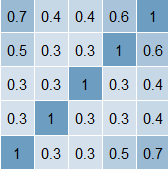 | 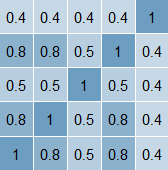 | 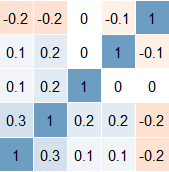 |
| RCA |  |  |  |  |  |  |  |  |  |
| RACMO |  |  |  |  |  |  |  |  |  |
| HIRAHM |  |  |  |  |  |  |  |  |  |
| CCLM |  |  |  |  |  |  |  |  |  |
|  | **CDD** | | | | | **CWM** | **HWM** | **PRCP TOT** | **r20mm** |
| 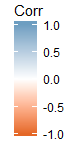 | 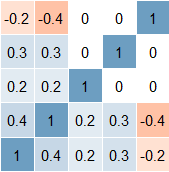 | | | | | 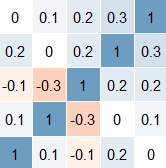 | 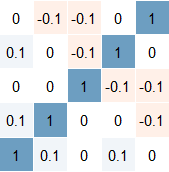 | 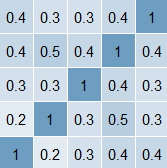 | 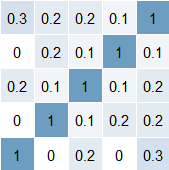 |
|  | **r95p** | | | | | **rJJA** | **rx1day** | **SPI-12E** | **SPI-3E** |

**Supplementary figure 2:** Spearman pairwise correlations between the standardised anomalies of selected extreme climate indices (abbreviations as in table 1 in the manuscript) for period 2071-2100, significant at 95% confidence level.

Table S3: Results of the analysis; index of the potential climate change impacts (CPI) obtained from SAW and OWA aggregation, and the adaptive capacity index ACI (32) by provinces (NUTS3 regions according to the Classification of Territorial Units for Statistics.

|  |  |  |  | **CPI scores** | | | | | | | **CPI OWA scores** | | | | | | | **ACI** |
| --- | --- | --- | --- | --- | --- | --- | --- | --- | --- | --- | --- | --- | --- | --- | --- | --- | --- | --- |
|  |  |  |  | **2021-2050** | | | **2071-2100** | | | | **2021-2050** | | | **2071-2100** | | | |  |
| ISTAT Code | Abbr | Name | NUTS3 code | **MAX** | **MIN** | **MED** | **MAX** | **MIN** | **MED** | **MAX** | | **MIN** | **MED** | | **MAX** | **MIN** | **MED** |  |
| 1 | TO | Torino | ITC11 | 0,230 | -0,302 | -0,104 | 0,177 | -0,538 | 0,028 | -0,110 | | -0,501 | -0,363 | | 0,062 | -0,733 | -0,543 | 0,436 |
| 2 | VC | Vercelli | ITC12 | 0,168 | -0,160 | -0,024 | 0,121 | -0,323 | -0,100 | 0,111 | | -0,341 | -0,249 | | 0,163 | -0,354 | -0,062 | 0,000 |
| 3 | NO | Novara | ITC15 | 0,190 | -0,117 | 0,075 | 0,144 | -0,290 | -0,042 | 0,172 | | -0,263 | -0,156 | | 0,191 | -0,313 | 0,008 | 0,416 |
| 4 | CN | Cuneo | ITC16 | 0,348 | -0,414 | 0,006 | 0,226 | -0,770 | -0,005 | -0,068 | | -0,295 | -0,073 | | 0,059 | -0,839 | -0,565 | 0,306 |
| 5 | AT | Asti | ITC17 | 0,134 | -0,112 | 0,023 | 0,141 | -0,354 | 0,040 | 0,115 | | -0,280 | -0,177 | | 0,117 | -0,270 | -0,106 | -0,082 |
| 6 | AL | Alessandria | ITC18 | 0,109 | -0,032 | 0,036 | 0,218 | -0,179 | -0,116 | -0,002 | | -0,193 | -0,123 | | -0,043 | -0,165 | -0,139 | 0,055 |
| 7 | AO | Aosta | ITC20 | -0,059 | -0,175 | -0,068 | 0,222 | -0,315 | -0,075 | -0,285 | | -0,367 | -0,287 | | -0,090 | -0,266 | -0,145 | 0,640 |
| 8 | IM | Imperia | ITC31 | 0,094 | -0,027 | -0,008 | 0,070 | -0,226 | -0,124 | 0,094 | | -0,255 | -0,170 | | 0,099 | -0,361 | -0,145 | -0,100 |
| 9 | SV | Savona | ITC32 | 0,110 | -0,109 | -0,043 | 0,035 | -0,253 | 0,022 | 0,098 | | -0,283 | -0,215 | | 0,085 | -0,261 | -0,154 | 0,339 |
| 10 | GE | Genova | ITC33 | 0,113 | -0,077 | 0,072 | 0,204 | -0,154 | 0,005 | 0,067 | | -0,194 | -0,098 | | 0,144 | -0,056 | 0,089 | 0,651 |
| 11 | SP | La Spezia | ITC34 | 0,187 | -0,095 | 0,055 | 0,150 | -0,270 | 0,055 | 0,169 | | -0,252 | -0,096 | | 0,201 | -0,246 | 0,113 | 0,474 |
| 12 | VA | Varese | ITC41 | 0,147 | -0,026 | 0,048 | 0,103 | -0,148 | -0,018 | 0,076 | | -0,157 | -0,126 | | 0,110 | -0,140 | 0,078 | 0,747 |
| 13 | CO | Como | ITC42 | 0,133 | -0,080 | 0,063 | 0,088 | -0,223 | 0,003 | 0,073 | | -0,193 | -0,116 | | 0,065 | -0,161 | 0,008 | 0,450 |
| 14 | SO | Sondrio | ITC44 | 0,265 | -0,210 | 0,069 | 0,292 | -0,273 | -0,086 | -0,135 | | -0,361 | -0,214 | | -0,041 | -0,196 | -0,146 | 0,201 |
| 15 | MI | Milano | ITC4C | 0,230 | 0,041 | 0,167 | 0,143 | -0,062 | -0,020 | 0,150 | | -0,086 | 0,035 | | 0,178 | -0,119 | 0,104 | 1,572 |
| 16 | BG | Bergamo | ITC46 | 0,158 | -0,178 | 0,065 | 0,122 | -0,222 | -0,140 | -0,095 | | -0,271 | -0,110 | | -0,033 | -0,270 | -0,073 | 0,568 |
| 17 | BS | Brescia | ITC47 | 0,683 | -0,491 | 0,196 | 0,976 | -0,494 | -0,340 | 0,189 | | -0,422 | 0,093 | | 0,143 | -0,447 | 0,015 | 0,338 |
| 18 | PV | Pavia | ITC48 | 0,224 | -0,110 | 0,100 | -0,015 | -0,241 | -0,204 | 0,061 | | -0,229 | -0,051 | | -0,008 | -0,333 | -0,128 | 0,388 |
| 19 | CR | Cremona | ITC4A | 0,247 | -0,022 | 0,071 | 0,145 | -0,431 | -0,049 | 0,186 | | -0,165 | -0,051 | | 0,081 | -0,548 | -0,165 | 0,324 |
| 20 | MN | Mantova | ITC4B | 0,252 | -0,030 | 0,079 | 0,114 | -0,656 | -0,108 | 0,038 | | -0,153 | -0,044 | | -0,024 | -0,632 | -0,262 | 0,364 |
| 21 | BZ | Bolzano | ITH10 | 1,000 | -1,000 | 0,469 | 1,000 | -1,000 | 0,081 | 0,347 | | -0,964 | 0,270 | | 0,319 | -0,941 | -0,075 | 1,139 |
| 22 | TN | Trento | ITH20 | 0,316 | -0,759 | -0,035 | 0,678 | -1,000 | -0,061 | -0,017 | | -1,000 | -0,298 | | 0,176 | -1,000 | -0,941 | 0,885 |
| 23 | VR | Verona | ITH31 | 0,464 | -0,109 | 0,084 | 0,540 | -0,521 | -0,045 | 0,080 | | -0,094 | 0,066 | | -0,031 | -0,344 | -0,143 | 0,513 |
| 24 | VI | Vicenza | ITH32 | 0,113 | -0,050 | -0,009 | 0,172 | -0,319 | -0,043 | -0,041 | | -0,230 | -0,180 | | -0,145 | -0,364 | -0,246 | 0,716 |
| 25 | BL | Belluno | ITH33 | -0,163 | -0,350 | -0,277 | -0,028 | -0,598 | -0,263 | -0,313 | | -0,759 | -0,717 | | -0,193 | -1,000 | -0,622 | 0,483 |
| 26 | TV | Treviso | ITH34 | 0,100 | -0,054 | 0,020 | 0,114 | -0,428 | -0,182 | -0,078 | | -0,147 | -0,109 | | -0,128 | -0,345 | -0,279 | 0,646 |
| 27 | VE | Venezia | ITH35 | 0,114 | -0,133 | 0,057 | 0,043 | -0,401 | -0,233 | -0,003 | | -0,143 | -0,088 | | -0,036 | -0,467 | -0,224 | 0,449 |
| 28 | PD | Padova | ITH36 | 0,146 | 0,024 | 0,069 | 0,094 | -0,423 | -0,111 | -0,014 | | -0,058 | -0,036 | | -0,057 | -0,333 | -0,215 | 0,744 |
| 29 | RO | Rovigo | ITH37 | 0,164 | -0,074 | 0,050 | 0,145 | -0,302 | -0,223 | 0,127 | | -0,199 | -0,102 | | 0,133 | -0,454 | -0,147 | -0,021 |
| 30 | UD | Udine | ITH42 | 0,158 | -0,095 | 0,030 | 0,151 | -0,183 | -0,095 | -0,078 | | -0,258 | -0,184 | | 0,087 | -0,203 | -0,077 | 0,359 |
| 31 | GO | Gorizia | ITH43 | 0,261 | -0,148 | 0,027 | 0,178 | -0,482 | -0,001 | 0,216 | | -0,293 | -0,134 | | 0,240 | -0,390 | 0,047 | 0,490 |
| 32 | TS | Trieste | ITH44 | 0,263 | -0,156 | 0,024 | 0,189 | -0,462 | 0,018 | 0,219 | | -0,295 | -0,145 | | 0,256 | -0,342 | 0,049 | 1,196 |
| 33 | PC | Piacenza | ITH51 | 0,130 | -0,024 | 0,053 | -0,028 | -0,204 | -0,122 | -0,014 | | -0,159 | -0,045 | | -0,039 | -0,242 | -0,198 | 0,388 |
| 34 | PR | Parma | ITH52 | 0,284 | -0,104 | 0,097 | 0,075 | -0,261 | -0,136 | 0,036 | | -0,131 | -0,003 | | -0,167 | -0,361 | -0,183 | 0,791 |
| 35 | RE | Reggio nell'Emilia | ITH53 | 0,265 | -0,012 | 0,146 | -0,023 | -0,165 | -0,127 | 0,027 | | -0,066 | 0,013 | | -0,113 | -0,231 | -0,201 | 0,679 |
| 36 | MO | Modena | ITH54 | 0,195 | 0,010 | 0,091 | 0,040 | -0,194 | -0,082 | 0,045 | | -0,098 | 0,002 | | -0,131 | -0,210 | -0,134 | 0,724 |
| 37 | BO | Bologna | ITH55 | 0,267 | -0,134 | 0,132 | 0,230 | -0,324 | 0,022 | 0,131 | | -0,262 | 0,121 | | 0,106 | -0,292 | -0,210 | 1,267 |
| 38 | FE | Ferrara | ITH56 | 0,188 | 0,020 | 0,079 | 0,071 | -0,356 | -0,126 | -0,008 | | -0,045 | -0,031 | | -0,051 | -0,390 | -0,306 | 0,271 |
| 39 | RA | Ravenna | ITH57 | 0,180 | -0,017 | 0,063 | 0,067 | -0,362 | -0,141 | -0,006 | | -0,058 | -0,012 | | -0,004 | -0,281 | -0,085 | 0,603 |
| 40 | FC | Forlì-Cesena | ITH58 | 0,197 | -0,050 | 0,000 | -0,010 | -0,155 | -0,048 | 0,014 | | -0,132 | -0,047 | | -0,027 | -0,104 | -0,051 | 0,618 |
| 41 | PU | Pesaro e Urbino | ITI31 | 0,225 | -0,157 | 0,004 | 0,365 | -0,144 | -0,094 | -0,019 | | -0,154 | -0,049 | | 0,036 | -0,098 | -0,051 | 0,384 |
| 42 | AN | Ancona | ITI32 | 0,204 | -0,057 | 0,023 | 0,060 | -0,155 | -0,085 | -0,057 | | -0,089 | -0,082 | | -0,040 | -0,131 | -0,118 | 0,602 |
| 43 | MC | Macerata | ITI33 | 0,325 | -0,064 | 0,003 | 0,205 | -0,167 | -0,099 | -0,060 | | -0,112 | -0,066 | | -0,032 | -0,160 | -0,099 | 0,311 |
| 44 | AP | Ascoli Piceno | ITI34 | 0,134 | -0,105 | 0,041 | 0,095 | -0,296 | 0,048 | 0,083 | | -0,229 | -0,133 | | 0,110 | -0,169 | 0,022 | 0,405 |
| 45 | MS | Massa-Carrara | ITI11 | 0,174 | -0,127 | 0,095 | 0,119 | -0,277 | 0,077 | 0,136 | | -0,215 | -0,068 | | 0,167 | -0,202 | 0,062 | -0,195 |
| 46 | LU | Lucca | ITI12 | 0,133 | -0,102 | 0,099 | 0,060 | -0,128 | -0,002 | 0,042 | | -0,099 | -0,019 | | 0,060 | -0,110 | 0,013 | 0,328 |
| 47 | PT | Pistoia | ITI13 | 0,192 | -0,085 | 0,080 | 0,143 | -0,199 | -0,059 | 0,166 | | -0,143 | -0,018 | | 0,154 | -0,279 | 0,016 | 0,245 |
| 48 | FI | Firenze | ITI14 | 0,351 | -0,081 | 0,131 | 0,546 | -0,235 | 0,200 | 0,239 | | -0,097 | 0,130 | | 0,358 | -0,167 | 0,290 | 0,922 |
| 49 | LI | Livorno | ITI16 | 0,152 | -0,118 | 0,082 | 0,161 | -0,253 | 0,081 | 0,138 | | -0,184 | -0,079 | | 0,186 | -0,168 | 0,141 | 0,660 |
| 50 | PI | Pisa | ITI17 | 0,222 | -0,027 | 0,114 | 0,308 | -0,071 | 0,009 | 0,106 | | 0,016 | 0,017 | | 0,231 | 0,010 | 0,102 | 0,737 |
| 51 | AR | Arezzo | ITI18 | 0,252 | -0,123 | 0,001 | 0,380 | -0,222 | 0,076 | 0,014 | | -0,137 | -0,020 | | 0,202 | -0,144 | -0,006 | 0,285 |
| 52 | SI | Siena | ITI19 | 0,313 | -0,057 | 0,000 | 0,416 | -0,137 | -0,106 | 0,119 | | -0,130 | 0,065 | | 0,237 | -0,159 | 0,009 | 0,768 |
| 53 | GR | Grosseto | ITI1A | 0,327 | -0,096 | 0,160 | 0,245 | -0,164 | 0,114 | 0,223 | | -0,113 | 0,204 | | 0,306 | -0,108 | 0,285 | 0,078 |
| 54 | PG | Perugia | ITI21 | 0,433 | -0,191 | 0,326 | 0,479 | -0,327 | 0,275 | 0,425 | | -0,396 | 0,340 | | 0,610 | -0,422 | 0,307 | 0,267 |
| 55 | TR | Terni | ITI22 | 0,122 | -0,021 | 0,083 | 0,122 | -0,178 | 0,023 | 0,044 | | -0,104 | -0,014 | | 0,173 | -0,039 | 0,077 | 0,008 |
| 56 | VT | Viterbo | ITI41 | 0,255 | 0,121 | 0,132 | 0,030 | -0,210 | -0,015 | 0,153 | | 0,098 | 0,111 | | 0,033 | -0,072 | -0,005 | -0,214 |
| 57 | RI | Rieti | ITI42 | 0,321 | 0,010 | 0,259 | 0,234 | -0,112 | 0,037 | 0,278 | | -0,001 | 0,079 | | 0,272 | -0,034 | 0,163 | -0,211 |
| 58 | RM | Roma | ITI43 | 1,000 | -0,146 | 0,586 | 0,889 | -0,379 | -0,237 | 1,000 | | -0,205 | 1,000 | | 0,864 | -0,555 | 0,450 | 0,838 |
| 59 | LT | Latina | ITI44 | 0,243 | 0,130 | 0,178 | 0,124 | -0,402 | -0,013 | 0,149 | | 0,057 | 0,113 | | 0,071 | -0,224 | 0,024 | -0,237 |
| 60 | FR | Frosinone | ITI45 | 0,273 | 0,015 | 0,135 | 0,163 | -0,192 | 0,058 | 0,137 | | -0,058 | 0,133 | | 0,196 | -0,169 | -0,111 | -0,531 |
| 61 | CE | Caserta | ITF31 | 0,520 | 0,127 | 0,341 | 0,111 | -0,217 | 0,055 | 0,328 | | 0,233 | 0,303 | | 0,133 | -0,036 | 0,029 | -0,775 |
| 62 | BN | Benevento | ITF32 | 0,322 | 0,083 | 0,146 | 0,204 | -0,101 | -0,013 | 0,112 | | 0,009 | 0,082 | | 0,294 | 0,000 | 0,083 | -0,685 |
| 63 | NA | Napoli | ITF33 | 0,420 | 0,196 | 0,336 | 0,137 | -0,148 | 0,103 | 0,288 | | 0,215 | 0,273 | | 0,280 | 0,059 | 0,072 | -0,728 |
| 64 | AV | Avellino | ITF34 | 0,220 | 0,037 | 0,158 | 0,282 | -0,077 | 0,108 | 0,056 | | -0,095 | 0,030 | | 0,238 | -0,034 | 0,174 | -0,652 |
| 65 | SA | Salerno | ITF35 | 0,864 | -0,147 | 0,536 | 0,928 | -0,268 | -0,046 | 0,822 | | -0,208 | 0,582 | | 0,767 | -0,350 | 0,229 | -0,400 |
| 66 | AQ | L'Aquila | ITF11 | 0,659 | -0,448 | 0,422 | 1,000 | -0,541 | 0,545 | 0,520 | | -0,397 | 0,490 | | 0,818 | -0,383 | 0,446 | 0,040 |
| 67 | TE | Teramo | ITF12 | 0,133 | -0,118 | 0,012 | 0,151 | -0,177 | -0,057 | -0,069 | | -0,135 | -0,108 | | 0,004 | -0,077 | -0,012 | 0,213 |
| 68 | PE | Pescara | ITF13 | 0,140 | -0,086 | 0,036 | 0,143 | -0,208 | 0,038 | 0,074 | | -0,172 | -0,097 | | 0,146 | -0,136 | 0,089 | 0,249 |
| 69 | CH | Chieti | ITF14 | 0,170 | -0,016 | 0,032 | 0,149 | -0,073 | 0,046 | -0,056 | | -0,083 | -0,080 | | 0,060 | 0,005 | 0,043 | 0,022 |
| 70 | CB | Campobasso | ITF22 | 0,172 | 0,000 | 0,090 | 0,127 | -0,182 | 0,024 | 0,039 | | -0,057 | -0,023 | | 0,075 | -0,040 | 0,024 | -0,584 |
| 71 | FG | Foggia | ITF46 | 0,746 | -0,033 | 0,259 | 0,193 | -0,489 | 0,127 | 0,441 | | -0,119 | 0,281 | | 0,141 | -0,157 | 0,107 | -0,762 |
| 72 | BA | Bari | ITF47 | 0,493 | -0,035 | 0,342 | 0,399 | -0,440 | 0,133 | 0,304 | | 0,158 | 0,171 | | 0,352 | 0,184 | 0,200 | -0,381 |
| 73 | TA | Taranto | ITF43 | 0,324 | 0,033 | 0,201 | 0,381 | -0,356 | -0,031 | 0,298 | | 0,080 | 0,123 | | 0,309 | 0,199 | 0,241 | -0,546 |
| 74 | BR | Brindisi | ITF44 | 0,394 | -0,068 | 0,165 | 0,348 | -0,391 | -0,028 | 0,339 | | -0,086 | 0,004 | | 0,302 | -0,045 | 0,190 | -0,778 |
| 75 | LE | Lecce | ITF45 | 0,596 | -0,061 | 0,223 | 0,440 | -0,168 | 0,103 | 0,417 | | 0,082 | 0,127 | | 0,320 | 0,112 | 0,266 | -0,801 |
| 76 | PZ | Potenza | ITF51 | 0,479 | -0,407 | -0,126 | 0,643 | -0,445 | 0,176 | 0,260 | | -0,699 | -0,129 | | 0,596 | -0,483 | -0,429 | -0,519 |
| 77 | MT | Matera | ITF52 | 0,192 | -0,094 | 0,110 | 0,123 | -0,059 | 0,008 | 0,069 | | -0,052 | 0,043 | | 0,146 | 0,007 | 0,106 | -0,751 |
| 78 | CS | Cosenza | ITF61 | 0,773 | -0,311 | -0,001 | 0,935 | -0,381 | -0,073 | 0,570 | | -0,422 | 0,006 | | 0,772 | -0,727 | -0,385 | -0,969 |
| 79 | CZ | Catanzaro | ITF63 | 0,256 | 0,001 | 0,098 | 0,208 | -0,092 | 0,017 | 0,150 | | -0,078 | -0,007 | | 0,191 | -0,042 | 0,098 | -1,080 |
| 80 | RC | Reggio di Calabria | ITF65 | 0,600 | 0,134 | 0,400 | 0,570 | 0,110 | 0,330 | 0,499 | | 0,256 | 0,378 | | 0,723 | 0,343 | 0,384 | -0,952 |
| 81 | TP | Trapani | ITG11 | 0,323 | 0,090 | 0,191 | 0,276 | -0,095 | 0,071 | 0,195 | | 0,023 | 0,076 | | 0,217 | 0,106 | 0,116 | -1,076 |
| 82 | PA | Palermo | ITG12 | 1,000 | -0,087 | 0,193 | 1,000 | -0,060 | 0,190 | 0,840 | | -0,008 | 0,350 | | 1,000 | 0,101 | 0,201 | -1,150 |
| 83 | ME | Messina | ITG13 | 0,566 | 0,080 | 0,126 | 0,429 | -0,053 | 0,032 | 0,362 | | 0,026 | 0,129 | | 0,361 | -0,191 | 0,076 | -0,819 |
| 84 | AG | Agrigento | ITG14 | 0,482 | 0,092 | 0,137 | 0,280 | -0,011 | 0,154 | 0,255 | | 0,105 | 0,221 | | 0,354 | 0,222 | 0,234 | -1,670 |
| 93 | PN | Pordenone | ITH41 | 0,098 | -0,106 | -0,026 | 0,054 | -0,304 | -0,122 | -0,058 | | -0,272 | -0,262 | | -0,009 | -0,334 | -0,192 | 0,920 |
| 94 | IS | Isernia | ITF21 | 0,124 | -0,045 | 0,046 | 0,183 | -0,259 | 0,031 | 0,079 | | -0,171 | -0,084 | | 0,129 | -0,106 | 0,125 | -0,551 |
| 96 | BI | Biella | ITC13 | 0,252 | -0,170 | -0,061 | 0,165 | -0,455 | -0,003 | 0,173 | | -0,342 | -0,223 | | 0,239 | -0,363 | -0,002 | 0,155 |
| 97 | LC | Lecco | ITC43 | 0,158 | -0,140 | 0,020 | 0,098 | -0,327 | 0,025 | 0,101 | | -0,275 | -0,158 | | 0,124 | -0,226 | -0,013 | 0,573 |
| 98 | LO | Lodi | ITC49 | 0,206 | -0,173 | 0,088 | 0,165 | -0,366 | -0,157 | 0,235 | | -0,283 | -0,112 | | 0,201 | -0,433 | -0,030 | 0,280 |
| 99 | RN | Rimini | ITH59 | 0,166 | -0,093 | 0,032 | 0,108 | -0,323 | 0,003 | 0,088 | | -0,199 | -0,091 | | 0,127 | -0,245 | 0,018 | 0,571 |
| 100 | PO | Prato | ITI15 | 0,247 | -0,135 | 0,034 | 0,165 | -0,388 | 0,040 | 0,204 | | -0,256 | -0,103 | | 0,234 | -0,278 | 0,087 | 0,450 |
| 101 | KR | Crotone | ITF62 | 0,217 | -0,037 | -0,004 | 0,216 | -0,187 | -0,090 | 0,162 | | -0,150 | -0,066 | | 0,187 | -0,105 | -0,079 | -1,525 |
| 102 | VV | Vibo Valentia | ITF64 | 0,241 | -0,030 | 0,059 | 0,236 | -0,261 | 0,016 | 0,196 | | -0,181 | -0,092 | | 0,254 | -0,146 | 0,072 | -1,220 |
| 103 | VB | Verbano-Cusio-Ossola | ITC14 | 0,159 | -0,245 | -0,185 | 0,072 | -0,426 | -0,113 | -0,062 | | -0,411 | -0,372 | | 0,078 | -0,296 | -0,185 | 0,219 |
| 108 | MB | Monza e della Brianza | ITC4D | 0,234 | -0,085 | 0,052 | 0,195 | -0,310 | 0,034 | 0,210 | | -0,251 | -0,110 | | 0,208 | -0,246 | 0,110 | 0,877 |
| 109 | FM | Fermo | ITI35 | 0,195 | -0,146 | 0,030 | 0,118 | -0,331 | 0,005 | 0,146 | | -0,246 | -0,139 | | 0,174 | -0,230 | 0,058 | 0,310 |
| 110 | BT | Barletta-Andria-Trani | ITF48 | 0,318 | 0,010 | 0,114 | 0,270 | -0,252 | -0,161 | 0,242 | | -0,087 | -0,002 | | 0,234 | -0,168 | 0,065 | -0,749 |
| 85 | CL | Caltanissetta | ITG15 | 0,346 | -0,035 | 0,143 | 0,280 | -0,079 | 0,082 | 0,242 | | 0,045 | 0,055 | | 0,268 | 0,169 | 0,219 | -1,477 |
| 86 | EN | Enna | ITG16 | 0,407 | -0,025 | 0,074 | 0,249 | -0,068 | 0,111 | 0,137 | | 0,064 | 0,136 | | 0,215 | 0,144 | 0,212 | -1,463 |
| 87 | CT | Catania | ITG17 | 0,780 | -0,094 | 0,306 | 0,474 | -0,274 | 0,255 | 0,474 | | 0,200 | 0,309 | | 0,651 | 0,178 | 0,293 | -0,866 |
| 88 | RG | Ragusa | ITG18 | 0,460 | 0,184 | 0,261 | 0,479 | 0,184 | 0,362 | 0,479 | | 0,096 | 0,230 | | 0,521 | 0,440 | 0,445 | -1,075 |
| 89 | SR | Siracusa | ITG19 | 0,469 | 0,137 | 0,247 | 0,347 | 0,060 | 0,171 | 0,492 | | 0,086 | 0,231 | | 0,357 | 0,136 | 0,283 | -1,140 |
| 90 | SS | Sassari | ITG25 | 0,423 | -0,005 | 0,170 | 0,510 | -0,105 | 0,191 | 0,273 | | -0,006 | 0,233 | | 0,506 | 0,069 | 0,433 | -0,667 |
| 91 | NU | Nuoro | ITG26 | 0,374 | -0,008 | 0,133 | 0,268 | -0,075 | 0,084 | 0,191 | | -0,037 | 0,144 | | 0,263 | 0,043 | 0,231 | -0,627 |
| 92 | CA | Cagliari | ITG27 | 0,302 | -0,007 | 0,175 | 0,289 | -0,030 | 0,106 | 0,147 | | 0,065 | 0,087 | | 0,346 | 0,096 | 0,243 | -0,286 |
| 95 | OR | Oristano | ITG28 | 0,376 | 0,092 | 0,144 | 0,189 | 0,028 | 0,150 | 0,192 | | 0,123 | 0,158 | | 0,433 | 0,165 | 0,234 | -0,751 |
| 104 | OT | Olbia-Tempio | ITG29 | 0,219 | -0,084 | 0,096 | 0,194 | -0,166 | 0,023 | 0,086 | | -0,043 | 0,011 | | 0,127 | -0,040 | 0,039 | -0,646 |
| 105 | OG | Ogliastra | ITG2A | 0,170 | -0,036 | 0,086 | 0,133 | -0,261 | 0,020 | 0,136 | | -0,131 | -0,054 | | 0,176 | -0,096 | 0,060 | -0,637 |
| 106 | VS | Medio Campidano | ITG2B | 0,177 | 0,007 | 0,116 | 0,210 | -0,229 | 0,110 | 0,215 | | -0,105 | -0,022 | | 0,244 | -0,089 | 0,241 | -0,906 |
| 107 | CI | Carbonia-Iglesias | ITG2C | 0,181 | 0,001 | 0,084 | 0,191 | -0,262 | 0,096 | 0,185 | | -0,127 | -0,030 | | 0,219 | -0,084 | 0,206 | -0,689 |

| Hazard | Built and manufactured capital | Natural capital | Social capital | Economic capital |
| --- | --- | --- | --- | --- |
| Flood | $\boldsymbol{R95p}\dot{\boldsymbol{\times}}\boldsymbol{P2}\dot{\boldsymbol{\times}}\boldsymbol{CM}\mathbf{13}$ |  | $\boldsymbol{R95p}\dot{\boldsymbol{\times}}\boldsymbol{P2}\dot{\boldsymbol{\times}}\boldsymbol{CU}\mathbf{1}$ | $\boldsymbol{R95p}\dot{\boldsymbol{\times}} \boldsymbol{P2}\dot{\boldsymbol{\times}}\sum\left( \boldsymbol{CE}\mathbf{1+}\boldsymbol{CE}\mathbf{2+}\boldsymbol{CE}\mathbf{3} \right)$ |
| Landslide | $\boldsymbol{rx1d}\dot{\boldsymbol{\times}}\boldsymbol{WP}\dot{\boldsymbol{\times}}\boldsymbol{LS}\dot{\boldsymbol{\times}}\boldsymbol{CM}\mathbf{13}$ |  | $\boldsymbol{rx1d}\dot{\boldsymbol{\times}}\boldsymbol{WP}\dot{\boldsymbol{\times}}\boldsymbol{LS}\dot{\boldsymbol{\times}}\boldsymbol{CU}\mathbf{1}$ |  |
| Flash flood | $\boldsymbol{FFPI}\dot{\boldsymbol{\times}}\boldsymbol{r95}\dot{\boldsymbol{\times}}\boldsymbol{CM}\mathbf{13}$ |  | $\boldsymbol{FFPI}\dot{\boldsymbol{\times}}\boldsymbol{R95p}\dot{\boldsymbol{\times}}\boldsymbol{CU}\mathbf{1}$ |  |
| Drought |  | $\boldsymbol{CDD}\dot{\boldsymbol{\times}}\boldsymbol{CN}\mathbf{1}$ |  | $\boldsymbol{SPI3}\dot{\boldsymbol{\times}}\boldsymbol{CE}\mathbf{1}$ |
| Heatwave |  |  | $\boldsymbol{HW}\dot{\boldsymbol{\times}}\boldsymbol{CM}\mathbf{2}\boldsymbol{a}\dot{\boldsymbol{\times}}\boldsymbol{CU}\boldsymbol{1\times0.75}$ $\mathbf{+}\boldsymbol{HW}\dot{\boldsymbol{\times}}\boldsymbol{CM}\mathbf{2}\boldsymbol{b}\dot{\boldsymbol{\times}}\boldsymbol{CU}\boldsymbol{1\times0.25}$ $\mathbf{+}\boldsymbol{HW}\dot{\boldsymbol{\times}}\boldsymbol{CM}\mathbf{2}\boldsymbol{a}\dot{\boldsymbol{\times}}\boldsymbol{CU}\boldsymbol{2\times0.75}$ $\mathbf{+}\boldsymbol{HW}\dot{\boldsymbol{\times}}\boldsymbol{CM}\mathbf{2}\boldsymbol{b}\dot{\boldsymbol{\times}}\boldsymbol{CU}\boldsymbol{2\times0.25}$ |  |
| Cold wave |  |  | $\boldsymbol{CW}\dot{\boldsymbol{\times}}\boldsymbol{CM}\mathbf{2}\boldsymbol{a}\dot{\boldsymbol{\times}}\boldsymbol{CU}\boldsymbol{1\times0.5+}$ $\boldsymbol{CW}\dot{\boldsymbol{\times}}\boldsymbol{CM}\mathbf{2}\boldsymbol{b}\dot{\boldsymbol{\times}}\boldsymbol{CU}\boldsymbol{1\times0.5}$ |  |
| Water scarcity |  |  | $\boldsymbol{PP}\dot{\boldsymbol{\times}}\boldsymbol{CU}\mathbf{1}$ | $\boldsymbol{PP}\dot{\boldsymbol{\times}}\boldsymbol{CE}\mathbf{1}$ |
| Soil erosion |  | $\boldsymbol{R20}\dot{\boldsymbol{\times}}\boldsymbol{CN}\mathbf{3}$ |  |  |

**Supplementary Table S4:** Combination (spatial overlay/multiplication denoted as $\dot{\times}$) algorithms for combining **hazard** and *exposure* indicators into potential climate change impact (CPI) index.

**References**

1. Oppenheimer M, Campos M, Birkmann J, Luber G, O’Neill B, Takahashi K, et al. . Emergent Risks and Key Vulnerabilities. Risk. 2014;19:1.

2. Jurgilevich A, Räsänen A, Groundstroem F, Juhola S. A systematic review of dynamics in climate risk and vulnerability assessments. Environ Res Lett. 2017;12(1):13002.

3. Romieu E, Welle T, Schneiderbauer S, Pelling M, Vinchon C. Vulnerability assessment within climate change and natural hazard contexts: revealing gaps and synergies through coastal applications. Sustain Sci. 2010;5(2):159–70.

4. O’Brien K, Eriksen SEH, Schjolden A, Nygaard LP. What’s in a word? Conflicting interpretations of vulnerability in climate change research. CICERO Work Pap. 2004;

5. IPCC. Summary for policymakers - Special report on managing the risk of extreme events and disasters to advance climate change adaptation (SREX). Intergovernmental Panel on Climate Change; 2012.

6. Cardona O-D, van Aalst MK, Birkmann J, Fordham M, McGregor G, Mechler R. Determinants of risk: exposure and vulnerability. 2012;

7. IPCC. Climate Change 2007: impacts, adaptation and vulnerability: contribution of Working Group II to the fourth assessment report of the Intergovernmental Panel on Climate Change (Parry M.L., Canziani O.F., Palutikof J.P., van der Linden P.J. e Hanson C.E.). Cambridge University Press; 2007.

8. IPCC. Climate Change 1995: Impacts, Adaptations, and Mitigation of Climate Change: Scientific-Technical Analyses. 1996;

9. McCarthy JJ. Climate change 2001: impacts, adaptation, and vulnerability: contribution of Working Group II to the third assessment report of the Intergovernmental Panel on Climate Change. Cambridge University Press; 2001.

10. ESPON. ESPON CLIMATE-Climate Change and Territorial Effects on Regions and Local Economies. 2011;

11. Bizikova L, Bellali J, Habtezion Z, Diakhite M, Pinter L. IEA Training Manual Volum Two: Vulnerability and Impact assessment for Adaptation to Climate Change (VIA Module). United Nations Environ Program. 2009;

12. Brooks N. Vulnerability, risk and adaptation: A conceptual framework. Working paper, Tyndall Centre for Climate Change; 2003. 1-16 p.

13. Binita KC, Shepherd JM, Gaither CJ. Climate change vulnerability assessment in Georgia. Appl Geogr. 2015;62:62–74.

14. Smit B, Wandel J. Adaptation, adaptive capacity and vulnerability. Glob Environ Chang. 2006;16(3):282–92.

15. Turner B, Kasperson R, Matson P. A framework for vulnerability analysis in sustainability science. Proc Natl Acad Sci. 2003;100(14):8074–9.

16. (MATTM) M dell’Ambiente e della T del T e del M. Piano Nazionale di Adattamento ai Cambiamenti Climatici PNACC. 2017;

17. MATTM. Strategia Nazionale di Adattamento ai Cambiamenti Climatici [Internet]. Roma,: Ministero dell’Ambiente e della Tutela del Territorio e del Mare; 2014. Available from: http://www.minambiente.it/sites/default/files/archivio/allegati/clima/documento_SNAC.pdf

18. Castellari S, Venturini S, Ballarin Denti A, Bigano A, Bindi M, Bosello F, et al. Rapporto sullo stato delle conoscenze scientifiche su impatti, vulnerabilità ed adattamento ai cambiamenti climatici in Italia. Ministero dell’Ambiente e della Tutela del Territorio e del Mare, Roma. 2014.

19. Castellari, S.,Venturini, S., Pozzo, B.,Tellarini, G., Giordano F. Analisi della normativa Comunitaria e nazionale rilevante per gli impatti, la vulnerabilità e l’adattamento ai cambiamenti climatici. Minist dell’Ambiente e della Tutela del Territ e del Mare, Roma. 2014;

20. Castellari S, Venturini S, Giordano F, Ballarin Denti A, Bigano A, Bindi M, et al. Elementi per una Strategia Nazionale di Adattamento ai Cambiamenti Climatici [Internet]. Rome, Italy; 2014. Available from: http://www.minambiente.it/sites/default/files/archivio/allegati/clima/snacc_2014_elementi.pdf

21. Jacob D, Petersen J, Eggert B, Alias A, Christensen OB, Bouwer LM, et al. EURO-CORDEX: new high-resolution climate change projections for European impact research. Reg Environ Chang [Internet]. 2014;14(2):563–78. Available from: http://dx.doi.org/10.1007/s10113-013-0499-2

22. Panagos P, Borrelli P, Poesen J, Ballabio C, Lugato E, Meusburger K, et al. The new assessment of soil loss by water erosion in Europe. Environ Sci Policy [Internet]. 2015 Dec;54:438–47. Available from: http://www.sciencedirect.com/science/article/pii/S1462901115300654

23. Amadio M, Mysiak J, Marzi S. Dasymetric mapping of socio-economic exposure for flood risk assessment in Italy. Risk Anal.

24. Merigó JM, Gil-Lafuente AM. The induced generalized OWA operator. Inf Sci (Ny). 2009 Mar;179(6):729–41.

25. Yager RR. Families of OWA operators. Fuzzy Sets Syst. 1993 Oct;59(2):125–48.

26. Marzi S, Mysiak J, Santato S. Comparing Adaptive Capacity Index across scales: the case of Italy. J Environ Manage.

27. ISTAT. Indicatori territoriali per le politiche di sviluppo. 2015.

28. Eurostat. Eurostat Database. 2017.

29. ISTAT. ISTAT Database. 2017.

30. ESPON. The Territorial Dimension of Poverty and Social Exclusion in Europe (TiPSE). 2012.

31. Nifo A, Vecchione G. Do institutions play a role in skilled migration? The case of Italy. Reg Stud. 2014;48(10):1628–49.

32. Handbook on constructing composite indicators. Paris: OECD publications; 2008.

1. https://www.wcrp-climate.org/data-etccdi [↑](#footnote-ref-1)
2. ClimPACT2 obtained from https://github.com/ARCCSS-extremes/climpact2 [↑](#footnote-ref-2)
3. Data were accessed last on 15 March 2017 from the Earth System Grid Federation (ESGF) node https://esgf-data.dkrz.de/search/cordex-dkrz/ [↑](#footnote-ref-3)
4. CDO and NCO were obtained from <https://code.zmaw.de/projects/cdo> and <http://nco.sourceforge.net/> respectively (accessed on April 07, 2017). [↑](#footnote-ref-4)
5. Abbreviations as in Table 2 of the manuscript. [↑](#footnote-ref-5)
